# Complete genome sequence and mass spectrometry assist mining siderophore turnerbactin analogs in a non-model *Pseudoduganella* strain with biocontrol potential

**DOI:** 10.3389/fmicb.2026.1888443

**Published:** 2026-07-23

**Authors:** Xuan Luo, Xianhui Huang, Hongyun Cao, Xianping Bai, Xingyan Wang, Dan Xing, Xinbei Zhang, Wenkang Li, Haiping Ni, Jingwen Sun, Lin Zhong, Baohang Huang, Qing Sun, Qiang Tu, Huawei Xin, Hanna Chen

**Affiliations:** 1 School of Medicine, Linyi University, Linyi, China; 2 Helmholtz International Lab for Anti-infectives, State Key Laboratory of Microbial Technology, Shandong University, Qingdao, Shandong, China; 3 Shandong Provincial Key Laboratory of Field Crop Physiology, Ecology, and Efficient Production (Under Construction), Jinan, China; 4 Institute of Synthetic Biology Industry, Hunan University of Arts and Science, Changde, China

**Keywords:** bioactivity, biosynthetic gene cluster, heterologous expression, *Pseudoduganella*, *siderophores*, whole-genome sequencing

## Abstract

Plant-associated biocontrol bacteria play a pivotal role in advancing sustainable agriculture by minimizing the reliance on toxic chemical pesticides. The order *Burkholderiales* is an underexplored yet highly promising group of microorganisms, characterized by a broad ecological distribution and considerable potential for application in sustainable agricultural systems. In this study, we aimed to isolate and characterize a potential biocontrol strain belonging to *Burkholderiales*, with the goal of contributing to eco-friendly agricultural practices and mining previously unexploited secondary metabolites. A novel bacterial strain, *Pseudoduganella* sp. D-3-1, was isolated from Cangma Mountain and characterized to possess potent antifungal activity and a high iron-chelating capacity. Whole-genome sequencing revealed that its complete genome spans 6,875,892 bp and encodes 6035 predicted coding sequences (CDSs), including a complete violacein biosynthesis pathway. Genome mining uncovered a diverse array of secondary metabolite biosynthetic gene clusters (BGCs), notably a non-ribosomal peptide synthetase (NRPS) gene cluster, designated the pseudodubactin cluster, which encodes a catecholate-type siderophore structurally related to turnerbactin. Through high-resolution electrospray ionization mass spectrometry (HR-ESI-MS), gene knockout, heterologous expression, and bioinformatics analysis, four turnerbactin analogs (**1**-**4**) were identified for the first time from *Pseudoduganella* sp., including two novel compounds: pseudodubactin A (**2**), and pseudodubactin B (**4**). Additionally, HR-ESI-MS analysis detected two uncharacterized siderophores, designated as compound **5** and compound **6**, that are structurally distinct from the turnerbactin analogs. These findings indicate that *Pseudoduganella* sp. D-3-1 is a promising source of bioactive natural products, particularly violacein and diverse siderophores, highlighting its potential as a biological control agent for sustainable and eco-friendly agriculture.

## 1 Introduction

Plant pathogens pose a significant threat to global agricultural productivity, contributing to yield losses in several major crops worldwide (Savary et al., 2019). While chemical pesticides have historically provided effective disease management, their extensive use has raised serious concerns regarding environmental sustainability and public health. As a sustainable, eco-friendly, and non-toxic alternative, biological control has gained increasing attention for its potential to mitigate plant diseases without the harmful side effects associated with chemical inputs (El-Saadony et al., 2022). A wide range of well-characterized microorganisms—including *Bacillus, Pseudomonas, Lactobacillus*, and members of the *Actinobacteria*—are widely employed as biofertilizers and biological control agents (BCAs) in agriculture (Babalola, 2010; Alattas et al., 2024) In addition, other beneficial microbes such as *Acetobacter, Serratia, Paenibacillus, Burkholderia*, and *Rhodococcus* have demonstrated strong biopesticidal potential (Grady et al., 2016; Elshafie and Camele, 2021; El-Saadony et al., 2022; Abou Jaoude et al., 2023; Campos et al., 2024). These organisms suppress plant pathogens through the production of diverse bioactive compounds, including lipopeptides, bacteriocins, antibiotics, siderophores, biosurfactants, cell wall-degrading enzymes, and microbial volatile organic compounds, which disrupt the growth and metabolism of phytopathogens (Timofeeva et al., 2023).

Among these, members of the order *Burkholderiales* represent an underexplored yet highly promising group of microorganisms with wide ecological distribution and significant potential in sustainable agriculture. Several species within this order, including *Burkholderia* (e.g., *Burkholderia cenocepacia* that inhibits plant pathogenic fungi (Song et al., 2024), the plant endophyte *B. seminalis* which improves plant growth under salt stress (Hwang et al., 2025), and *B. gladioli* that suppresses fungal growth and promotes plant development (Wang et al., 2023)) and *Paraburkholderia* (e.g., *P. phytofirmans* which possesses plant growth-promoting capacity and can elicit plant resistance to both biotic and abiotic stresses (Esmaeel et al., 2018a), *P. fungorum* with against phytopathogenic viruses activity (Shahzad et al., 2022), and *P. oxyphila* that exerts antifungal effects and triggers plant systemic resistance (Poulaki et al., 2024), and nitrogen-fixing *P. caballeronis* (Rojas-Rojas et al., 2017), that make them strong candidates for application as biocontrol agents and plant growth promoters. These traits include nitrogen fixation, effective rhizosphere colonization, stress tolerance induction, and, notably, the production of diverse secondary metabolites (Esmaeel et al., 2018b; Pal et al., 2022). These secondary metabolites, including lipopeptides and siderophores, play essential roles in the ecological fitness of *Burkholderiales*, mediating complex interactions with inhibiting phytopathogens and promoting host plants.

Siderophores are low-molecular-weight organic compounds produced by microorganisms and plants under iron-limiting conditions (Hider and Kong, 2010). Beyond their primary role in iron acquisition, siderophores hold great potential in environmental, agricultural, and medical fields. They are being explored for bioremediation, enhancing soil nutrients, boosting crop yields, and advancing antimicrobial therapies, medical imaging, and cancer treatment (Prabhakar, 2020; Schalk, 2025). Structurally, siderophores are typically classified based on their iron-chelating functional groups into three major categories: catecholates, hydroxamates, and α-hydroxycarboxylates (Hider and Kong, 2010; Swayambhu et al., 2021). Their biosynthesis involves complex enzymatic machinery, most commonly non-ribosomal peptide synthetases (NRPSs), polyketide synthases (PKSs), and NRPS-independent synthetases. Among siderophore-producing microbes, the order *Burkholderiales* is particularly notable for its ability to thrive under iron-restricted conditions by synthesizing diverse siderophores, including caribactin, ornibactin, malleobactin, pyochelin, and cepaciachelin (Franke et al., 2013; Esmaeel et al., 2016, 2018b; Wang et al., 2023), many of which are produced through NRPS-dependent pathways. Pyochelin is consistently co-produced with ornibactin and malleobactin and has demonstrated antibacterial activity against certain strains (Esmaeel et al., 2018b). Ornibactin plays a key role in the virulence of *B. cenocepacia* (Visser et al., 2004), while caribactins has been shown to promote biofilm formation and enhance both swarming and swimming motility (Wang et al., 2023). In contrast, the physiological roles of malleobactin and cepaciachelin remain poorly understood. The structural diversity, broad bioactivity, and ecological importance of siderophores highlight *Burkholderiales* as a valuable reservoir of bioactive metabolites with promising applications in sustainable agriculture and biomedicine.

In this study, we isolated and characterized a novel strain, *Pseudoduganella* sp. D-3-1, identified through 16S rRNA gene and whole-genome sequencing analyses. *Pseudoduganella* sp. D-3-1 exhibited broad-spectrum antimicrobial activity and strong siderophore production as confirmed by CAS agar assay. Using HR-ESI-MS, gene knockout, heterologous expression, and bioinformatics, we identified two known turnerbactin analogs in *Pseudoduganella* sp. D-3-1—representing the first report of such compounds in this genus. Despite its recent establishment by Kämpfer et al. in 2012 (Kampfer et al., 2012), the *Pseudoduganella* genus remains poorly studied, particularly regarding its taxonomy and capacity for secondary metabolite production. Our findings highlight *Pseudoduganella* sp. D-3-1 as a promising source of bioactive natural products and a potential candidate for applications in biological control and biofertilization, contributing to the advancement of sustainable agricultural practices.

## 2 Materials and methods

### 2.1 Bacterial isolation and phylogenetic analysis

Strain D-3-1 was isolated from soil collected on the mountainside of Cangma Mountain in Linshu County, Linyi City, Shandong Province, China, using the serial dilution method. Soil samples were diluted to an appropriate concentration (10^−1^) and spread evenly onto actinomycete agar medium (or modified Gause's No. 1 medium) for cultivation. The taxonomic identification of strain D-3-1 was based on 16S rRNA gene sequencing and whole-genome analysis. Genomic relatedness was assessed using average nucleotide identity (ANI) and digital DNA-DNA hybridization (dDDH). ANI values were computed via the OrthoANI algorithm on the EzBioCloud web service (www.ezbiocloud.net/sw/oat) (Lee et al., 2016), whereas dDDH values were determined with the Genome-to-Genome Distance Calculator 3.0 (http://ggdc.dsmz.de/ggdc.php#) (Meier-Kolthoff et al., 2013). DNA-DNA hybridization (DDH) experiments were performed among strains D-3-1 with closely related strains. For routine cultivation, D-3-1 was grown on actinomycete culture medium and 0238 medium (Peptone 5.0 g L^−1^, Yeast extract 10 g L^−1^, MgSO_4_ 7H_2_O g L^−1^).

### 2.2 Preparation and analysis of crude fermentation extracts

Two methods were employed to obtain crude fermentation extracts from strain D-3-1. In the first method, strain D-3-1 was cultured in 50 mL of actinomycete culture medium in a 250 mL flask at 30 °C and 200 rpm for 3 days. After incubation, 2% (w/v) XAD-16 resin was added to the culture and allowed to incubate for an additional 24 h under the same conditions. The resin and bacterial cells were collected by centrifugation at 8,000 rpm for 5 min and extracted with 20 mL of methanol (MeOH) for 3 h. The methanolic extract was then evaporated to dryness and redissolved in 1 mL of MeOH.

In the second method, strain D-3-1 was also cultured in 50 mL of actinomycete culture medium (with or without 2 mM FeCl_3_ supplementation) in a 250 mL flask at 30 °C and 200 rpm for 3 days. After fermentation, the culture broth was acidified to pH 2–3 and extracted twice with an equal volume of ethyl acetate. The extracted phases of ethyl acetate were evaporated to dryness and then redissolved in 1 mL of MeOH.

The crude extracts of ethyl acetate were subjected to HR-ESI-MS, CAS agar assay, and antimicrobial assays. A Thermo Scientific™ Acclaim™ C18 column (2.1 × 100 mm, 2.2 μm) was used. HR-ESI-MS analysis was performed under the following gradient conditions: 1-3 min, 5% ACN; 3-18 min, 5% to 95% ACN; 18–22 min, 95% ACN; and 22.1–25 min, 5% ACN. The mobile phase used H_2_O with 0.1% FA and 0.1% FA in ACN with 0.3 mL/min flow rate. The base peak chromatogram (BPC) was recorded over the *m*/*z* range of 100–1500, with UV detection in the 100–400 nm range.

### 2.3 Construction of recombineering system in D-3-1 and knockout of *pdb* and *vio* BGCs

Strain D-3-1 and its mutant derivatives were cultured on 0238 medium (broth and agar) at 30 °C. Mutant strains were selected on media supplemented with 20 μg mL^−1^ kanamycin (Km), 20 μg mL^−1^ gentamicin (Genta), or 20 μg mL^−1^ apramycin (Apra). Genome editing is mediated by linear and circular homologous recombination. Strains and plasmids used in this study were shown in Supplementary Table 1, and primers used in this study were shown in Supplementary Table 2.

To establish an efficient recombineering system in *Pseudoduganella* sp. D-3-1, three recombination systems were evaluated: Redγ*βα*, Redγ-Redαβ7029, and Redγ-BAS (Wang et al., 2018; Zheng et al., 2020; Chen et al., 2021). The detailed process was according to the previous study of *Burkholderiales* DSM 7029 (Wang et al., 2018). Recombination efficiency was assessed by attempting to replace a 3,032 bp fragment (1,908,943–1,911,975) within the violacein (*vio*) biosynthetic gene cluster with a gentamicin resistance gene flanked by ~1 kb homology arms. This experiment was carried out in D-3-1 strains harboring the plasmids pBBR1-Rha-Redγ*βα*-Km, pBBR1-Rha-Redγ-BAS-Km, and pBBR1-Rha-Redγ-Redαβ7029-Km, respectively.

The upstream and downstream homology arms (~1 kb each) of the target gene cluster (*pdb*) were amplified using specific primer pairs. The apramycin resistance gene was amplified using the primer pair apra-primer-S/apra-primer-A. Subsequently, the three purified fragments were used as templates for fusion PCR with the primer pair PseBGC14KO-HA11-S/PseBGC14KO-HA11-A to obtain the final PCR product. The resulting fusion PCR products were introduced into *Pseudoduganella* sp. D-3-1 harboring the Redγ-BAS recombination system.

### 2.4 Direct cloning and heterologous expression of *pdb* BGC

The *pdb* BGC from *Pseudoduganella* sp. D-3-1 was directly cloned by ExoCET method resulting in the plasmid p15A-cm-*pdb*. The genomic DNA was digested with *Xba*I and *BstZ17*I for cloning the 35.3-kb *pdb* gene cluster. Using primer PseBGC14-clone-S1/PseBGC14-clone-A1 amplified the vector fragment p15A-cm1 and the template is plasmid p15A-cm-tetO-tetR-ccdB, then, the fragment p15A-cm1 as a template, we used another primer PseBGC14-clone-S2/PseBGC14-clone-A2 to obtain the final vector fragment p15A-cm2 with the homology arm 70 bp. Then, we amplified km-attP fragment through primer short-attP-S/short-attP-A, and obtained km-attP inserted into the construct p15A-cm-*pdb* yielding the final construct p15A-km-attP-*pdb*. The construct p15A-km-attP-*pdb* was transformed into *E. coli* MW3064, and then was transformed into the heterologous host *B. gladioli* ATCC 10248Δ*gbn*-attB. The correct recombinants were verified by colony PCR.

### 2.5 Antimicrobial assays

The antimicrobial activity of the fermentation crude extracts from strain D-3-1 was evaluated using the filter paper disk diffusion method. Four indicator strains—*Pseudomonas aeruginosa* ATCC 27853, *Bacillus subtilis* ATCC 6633, *Staphylococcus aureus* ATCC 29213, and *Escherichia coli* ATCC 35218—were evenly spread on LB plates, respectively. Sterile filter paper disks (6 mm diameter) were loaded with 10 μL of crude extract and placed onto the inoculated indicator strain plates. The plates were incubated at 37 °C for 16 h, after which zones of inhibition were examined to evaluate antibacterial activity.

*Fusarium solani* were inoculated at the center of PDA plate, and 10 microliters of each sample (either cultures of strain D-3-1 or crude extracts) was added to the wells. The plate was then incubated at 30 °C for 48 h, after which inhibition activity was assessed to evaluate antifungal activity.

### 2.6 CAS agar assay

Ten microliters of each sample (cultures of strain D-3-1 or crude extracts) was added to the wells of CAS plates. The diameter of the orange halo (siderophore-chelating zone) was observed to evaluate siderophore production. Data analysis and figure plotting were performed using Microsoft Office Excel. All data are presented as mean values ± SD, *n* = 3 biologically independent samples. All *P* values were determined by two-tailed unpaired t test. ^*^p < 0.05, ^**^p < 0.01, ^***^p < 0.001, ^****^p < 0.0001. The CAS detection medium was purchased from Qingdao Hope Bio-Technology Co. Ltd.

### 2.7 Growth curve measurement

The wild type strain D-3-1 and its mutants were individually inoculated into 15 mL of sterile 0238 liquid medium, followed by overnight incubation at 30 °C with shaking at 200 rpm to obtain seed cultures. Each overnight seed culture was then transferred into fresh sterile 0238 liquid medium, and the optical density at 600 nm (OD_600_) of each culture was adjusted to 0.1 using fresh 0238 liquid medium to standardize the initial inoculum density. All adjusted cultures were incubated at 30 °C with shaking at 200 rpm for continuous growth. During the incubation period, the OD_600_ values of each culture were measured at intervals of 3 h, 6 h, and 12 h, respectively. Each strain (wild type and mutants) was tested with three biological replicates.

## 3 Results

### 3.1 Characterization and genome sequence analysis of *Pseudoduganella* sp. D-3-1

**Figure 1:**
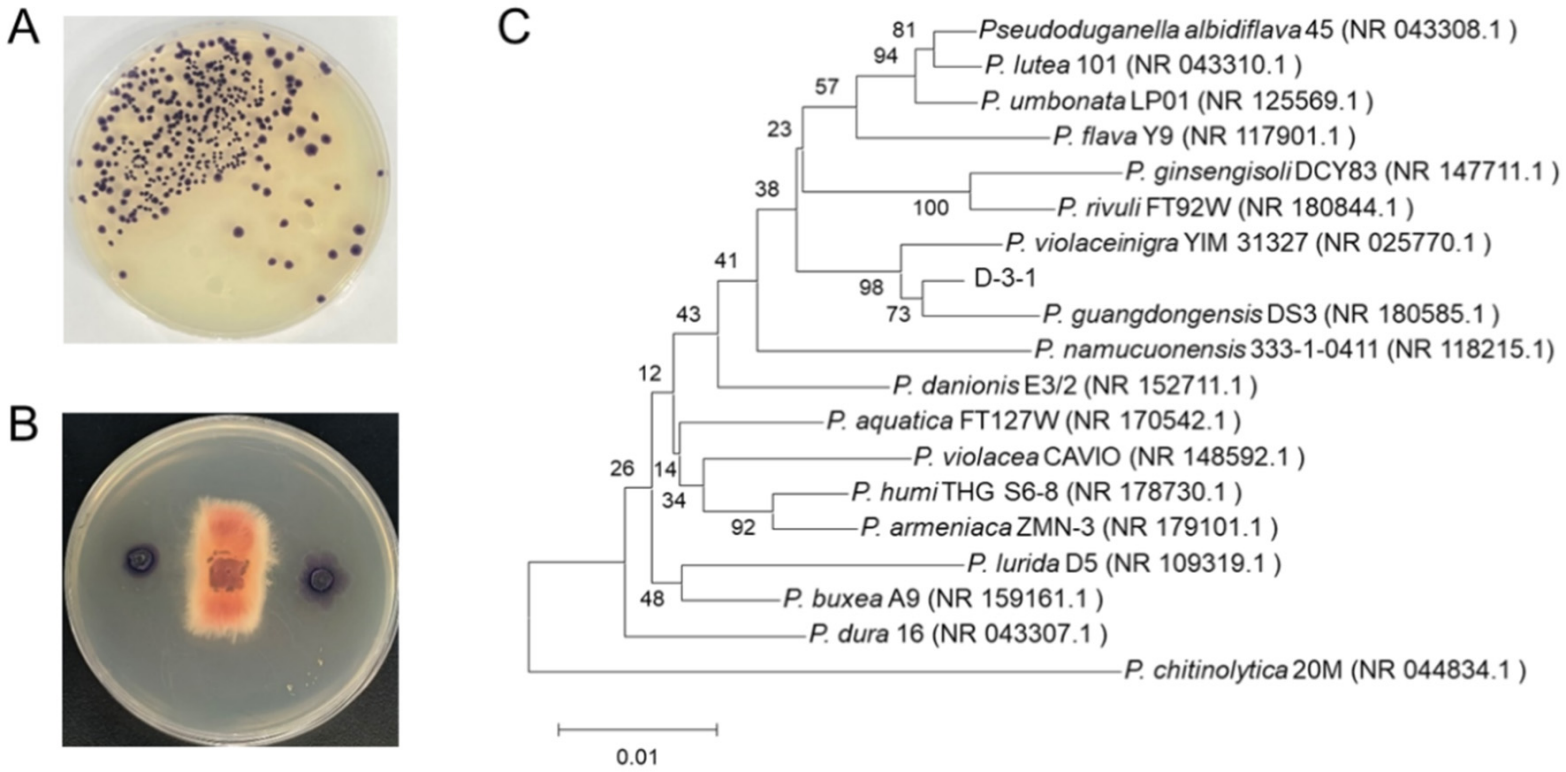
Colony morphology, antagonistic activity, and phylogenetic analysis of D-3-1 strain. **(A)** Colony morphology of D-3-1. **(B)** Antagonistic activity of strain D-3-1 against the *Fusarium solani*. **(C)** Phylogenetic tree of strain D-3-1 based on 16S rRNA sequence.

We isolated a microorganism from soil collected on the mountainside of Cangma Mountain in Linshu County Shandong Province. The isolate, designated D-3-1, is a Gram-negative bacterium that forms smooth, round, purple colonies on the actinomycetes agar medium (Figure 1A). *In vitro* assays demonstrated that strain D-3-1 exhibits strong antagonistic activity against *Fusarium solani* (Figure 1B). Using the D-3-1 culture as a template, the 16S rRNA gene was successfully amplified with primers 27F/1492R and subsequently sequenced. BLAST analysis was performed to identify 16S rRNA sequences with the highest similarity to that of D-3-1, and a phylogenetic tree was constructed using MEGA11 software (Figure 1C) (Tamura et al., 2021). However, the 16S rRNA-based phylogeny was insufficient to conclusively determine whether D-3-1 belongs to the genus *Pseudoduganella*. To further ascertain its taxonomy and investigate its antimicrobial potential, we sequenced the complete genome of strain D-3-1.

The genome of strain D-3-1 was sequenced using a combination of second-generation Illumina and third-generation Nanopore sequencing technologies. The assembled genome comprises 6,875,892 bp with a GC content of 62.88%, total 6194 genes, 22 rRNA, 87 tRNA, and contains a substantial proportion of genes with unknown functions (25.72%) (Table 1 and Supplementary Figures 1B, 1C). The top BLAST hits matched annotated unigenes from *Pseudoduganella violaceinigra, Duganella* sp. and *Massilia* sp. et al. The 16S rRNA gene sequence of strain D-3-1 shared 98.9% identity with the type strain *Pseudoduganella violaceinigra* DSM 15887^T^ (Kampfer et al., 2012). However, the average nucleotide identity (ANI, 88.2%) and digital DNA-DNA hybridization (dDDH, 36.6%) values were much lower than the thresholds of 95% and 70%, respectivley (Supplementary Table 4). Collectively, these genomic metrics demonstrate that strain D-3-1 represents a novel species belonging to the genus *Pseudoduganella*, which we tentatively designated *Pseudoduganella* sp. D-3-1. To further characterize the genome and identify potential antimicrobial biosynthetic pathways, we used antiSMASH (version 8.0.1) to predict secondary metabolite biosynthetic gene clusters (Blin et al., 2025). A total of 14 gene clusters were identified, including those potentially responsible for the biosynthesis of indole violacein, ralsolamycin, and the siderophore turnerbactin (Table 2).

**Table 1:** Genome features of *Pseudoduganella* sp. D-3-1.

Features	Value
Genome size (bps)	6,875,892
Contig	1
Chromosome	1
Plasmid	0
G+C content (%)	62.88
Total genes	6,194
rRNA	22
tRNA	87
Protein-coding genes (CDS)	6,035

**Table 2:** Secondary metabolite BGCs predicted in the genome of *Pseudoduganella* sp.

BGCs	Type	From	to	Similar cluster	Similarity confidence
BGC1	Acyl-amino acids	529,995	5,90,747	-	-
BGC2	RiPP-like	636,740	6,47,558	-	-
BGC3	Terpene-precursor	1,435,646	1,456,536	-	-
BGC4	indole	1,898,974	1,922,000	violacein	High
BGC5	RiPP-like	2,090,344	2,101,945	-	-
BGC6	Azole-containing RiPP	2,671,012	2,693,194	-	-
BGC7	Terpene	3,505,926	3,527,622	-	-
BGC8	Acyl-amino acids	3,550,678	3,611,391	-	^−^
BGC9	Azole-containing RiPP	3,702,298	3,733,359	-	-
BGC10	Arylpolyene	3,819,347	3,862,964	APE Vf	Low
BGC11	Trans-AT-PKS, NRPS	3,924,541	4,012,143	ralsolamycin	Low
BGC12	RiPP-like	4,304,990	4,315,871	-	-
BGC13	Acyl-amino acids	5,729,330	5,790,094	-	-
BGC14	NRPS-metallophore, NRPS	5,857,643	5,917,100	turnerbactin	Medium

Gene cluster predictions were generated using antiSMASH (version 8.0.1).

Violacein is a well-known indole derived violet pigmented compound with antibacterial, antifungal, antiprotozoan, and anticancer properties (Lyakhovchenko et al., 2022). Several bacterial genera are known producers of violacein, including *Chromobacterium violaceum, Janthionobacterium lividum, Massilia, Duganella*, and *Pseudoalteromonas* et al. (Ahmed et al., 2021; Park et al., 2021). The presence of a complete violacein biosynthetic pathway (BGC4) within the *Pseudoduganella* sp. D-3-1 genome suggests that this strain could serve as a promising alternative host for the industrial production of violacein. Turnerbactin, a catecholate siderophore, was originally identified in the shipworm endosymbiont *Teredinibacter turnerae* T7901 (Han et al., 2013). In strain D-3-1, a NRPS gene cluster (BGC14) associated with turnerbactin biosynthesis was detected, including four genes involved in the synthesis and activation of 2,3-dihydroxybenzoate (DHB). Due to their low similarity confidence, two additional gene clusters, BGC10 and BGC11, currently lack reference value.

### 3.2 Biological activity screening of secondary metabolites produced by *Pseudoduganella* sp. D-3-1

**Figure 2:**
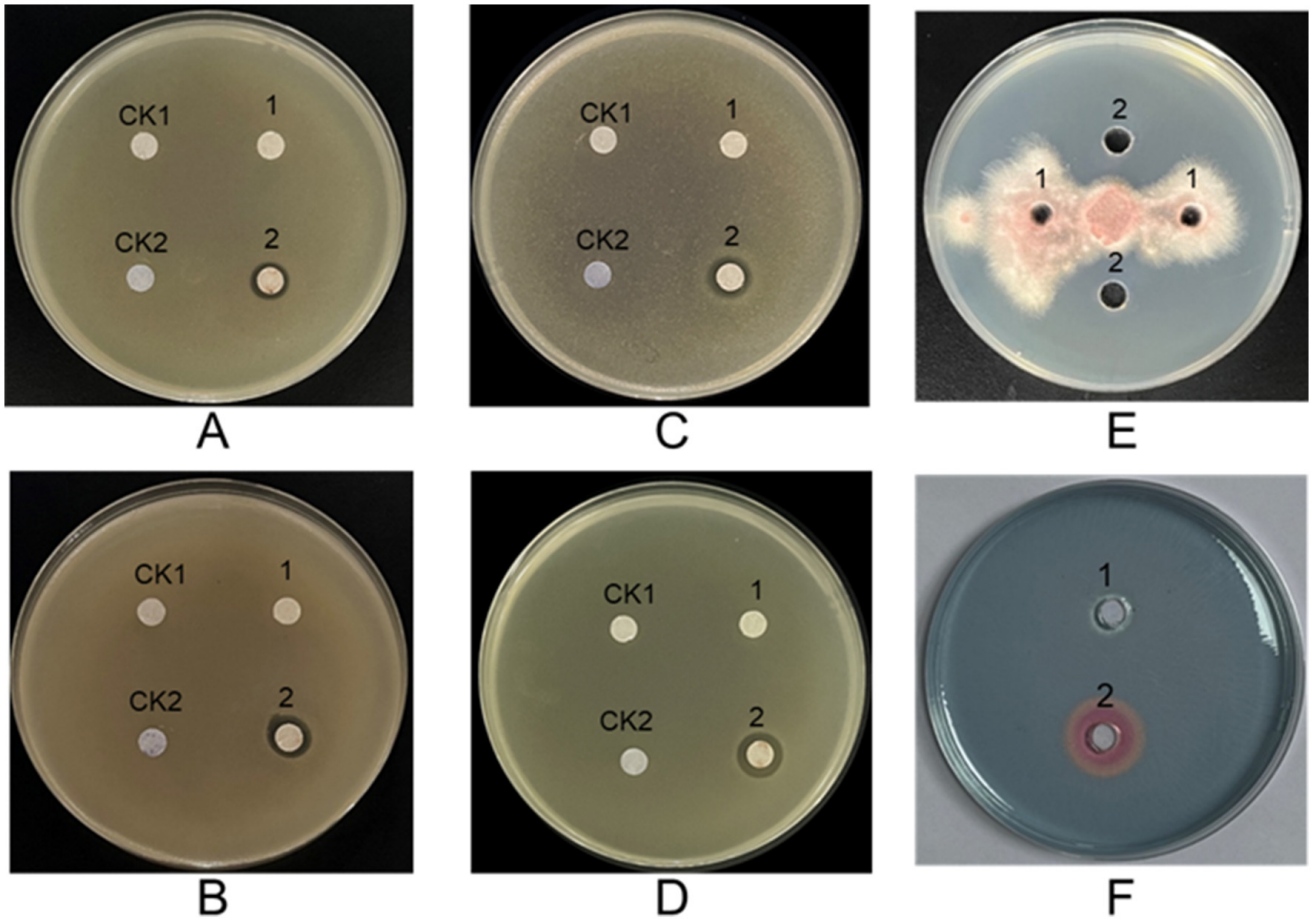
Siderophore activity and antimicrobial activity of crude extracts from strain D-3-1. **(A-D)** Antibacterial activity against *Staphylococcus aureus* **(A)**, *Bacillus subtilis* **(B)**, *Pseudomonas aeruginosa* PAO1 **(C)** and *Escherichia coli* **(D)**. **(E, F)** Antifungal activity against *Fusarium solani* **(E)**. Siderophore activity **(F)**. CK1: methanol crude extract of actinomycete liquid culture, 1: methanol crude extract of fermentation, CK2: ethyl acetate crude extract of actinomycete liquid culture, 2: ethyl acetate crude extract of fermentation.

To further investigate the diverse biological activities of secondary metabolites produced by *Pseudoduganella* sp. D-3-1, we applied the OSMAC (One Strain–Many Compounds) strategy (Zhang et al., 2024). Ethyl acetate crude extracts obtained from cultures grown in actinomycete liquid medium exhibited significant antifungal activity against *Fusarium solani*, as well as antibacterial activity against *B. subtilis, S. aureus, E. coli*, and *P. aeruginosa* (Figures 2A–E). In contrast, the methanol crude extracts showed no detectable antimicrobial activity. Additionally, siderophore production was assessed using the CAS agar assay, which confirmed that *Pseudoduganella* sp. D-3-1 is capable of producing siderophores (Figure 2F). Thus, the bioactive secondary metabolites are extractable using ethyl acetate, highlighting its effectiveness for obtaining active compounds from *Pseudoduganella* sp. D-3-1.

### 3.3 Characterization of turnerbactin-analog siderophores produced by *Pseudoduganella* sp. D-3-1

**Figure 3:**
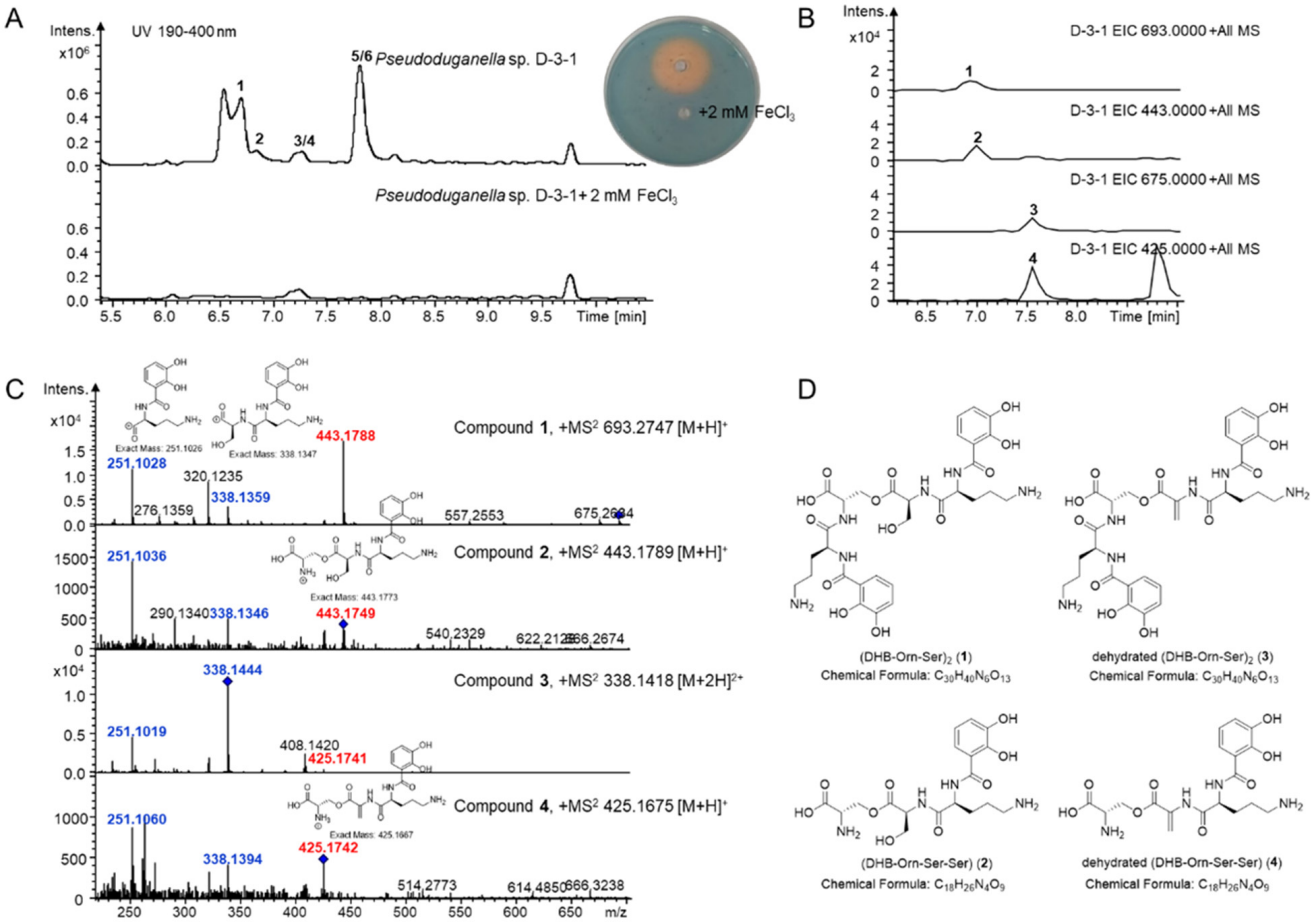
HR-ESI-MS analysis of the metabolic profile of *Pseudoduganella* sp. D-3-1 under iron-limited and iron-replete conditions and proposed structures of turnerbactin analogs. **(A)** Comparison of the metabolic profile of *Pseudoduganella* sp. D-3-1 cultured under iron-limited and iron-replete conditions at UV 190-400 nm and their CAS activity. **(B)** Extracted ion chromatograms (EIC) of compounds **1-4** detected at *m*/*z* 693, 443,443, 425, respectively. **(C)** MS/MS fragmentation patterns of compounds **1-4**. **(D)** Proposed chemical structures of turnerbactin analogs **1-4**.

To investigate siderophore production by *Pseudoduganella* sp. D-3-1, culture extracts were analyzed for the CAS activity and subjected to metabolic profiling under iron-limited and iron-replete conditions. The crude extract from iron-limited cultures exhibited strong CAS activity, whereas negligible activity was detected under iron-replete conditions (Figure 3A). Metabolic profiling revealed that at least three distinct peaks between 6-8 min were notably absent (Figure 3A). HR-ESI-MS analysis identified the following compounds: the molecular ion [*M*+H]^+^ of (DHB-Orn-Ser)_2_ (**1**), with *m*/*z* 693.2747, corresponding to the molecular formula of C_30_H_41_N_6_O_13_ (calculated *m*/*z* 693.2726); its dehydrated form (**3**), with *m*/*z* 675.2633, corresponding to a molecular formula of (C_30_H_41_N_6_O_13_, calculated *m*/*z* 675.2620); DHB-Orn-Ser-Ser (**2**), with *m*/*z* 443.1789, (C_18_H_27_N_4_O_9_, calculated 443.1773); and its dehydrated counterpart (**4**), with *m*/*z* 425.1675 (C_18_H_27_N_4_O_9_, calculated 425.1667) (Figure 3B).

MS/MS fragmentation patterns of compounds **1** and **2** revealed diagnostic fragments at *m*/*z* 251, 338, and 443, consistent with those of turnerbactin (Figure 3C) (Han et al., 2013). In contrast, compounds **3** and **4** displayed a distinct fragment at *m*/*z* 425 instead of 443, corresponding to a mass difference of 18 Da. This shift is consistent with the fragmentation patterns of those of dehydrated (DHB-Orn-Ser)_2_ and dehydrated (DHB-Orn-Ser)_3_ (Figure 3C) (Han et al., 2013). These fragmentations patterns confirm that compounds **1**-**4** are structural analogs of turnerbactin. However, authentic turnerbactin (DHB-Orn-Ser)_3_ with *m*/*z* 1030.4 (C_30_H_41_N_6_O_13_) was not detected in the crude extract. Structural comparison suggests that compounds **1**-**4** are likely hydrolysis products of turnerbactin, potentially resulting from acidic extraction conditions or premature release during biosynthesis (Han et al., 2013; Chen et al., 2020). Based on MS/MS data and bioinformatic analyses, we deduced the structures of four turnerbactin analogs (**1**-**4**), including two newly identified compounds- pseudodubactin A (**2**) and pseudodubactin B (**4**) (Figure 3D). Isolation of compounds **2** and **4** for NMR-based full structural elucidation proved unfeasible, owing to their low production titers and overlapping polarity with analogs **1** and **3**. These results indicate that the *tnb*-like gene cluster in *Pseudoduganella* sp. D-3-1, designated as the *pdb* gene cluster, is likely responsible for the biosynthesis of these turnerbactin analogs.

Notably, two uncharacterized siderophores, designated as compound **5** (*m*/*z* 390.0045) and compound **6** (*m*/*z* 169.0485), was detected by HR-ESI-MS analysis and appeared structurally distinct from the turnerbactin analogs (Figure 3A and Supplementary Figure 2). MS/MS fragmentation of compound **5** revealed a prominent fragment at *m*/*z* 169, matching the molecular ion of compound **6** (Fig. S2B), suggesting that compound **6** might be a hydrolysis product of compound **5**. We sought to deduce their chemical scaffolds by integrating MS/MS fragmentation profiles and bioinformatic predictions. Unexpectedly, these siderophores are not NRPS-derived metabolites, which severely impedes reliable structural prediction. To investigate whether these compounds are synthesized independently of the *pdb* gene cluster, we performed targeted gene deletion of the *pdb* gene cluster via homologous recombination. Additionally, violacein (**7**, *m*/*z* 344.1022) and deoxyviolacein (**8**, *m*/*z* 328.1074) were detected in the metabolic profile of *Pseudoduganella* sp. D-3-1 (Supplementary Figure 3), highlighting its potential candidate for efficient biotechnological production of violacein.

### 3.4 Knockout of *pdb* and *vio* BGCs using a recombineering system

**Figure 4:**
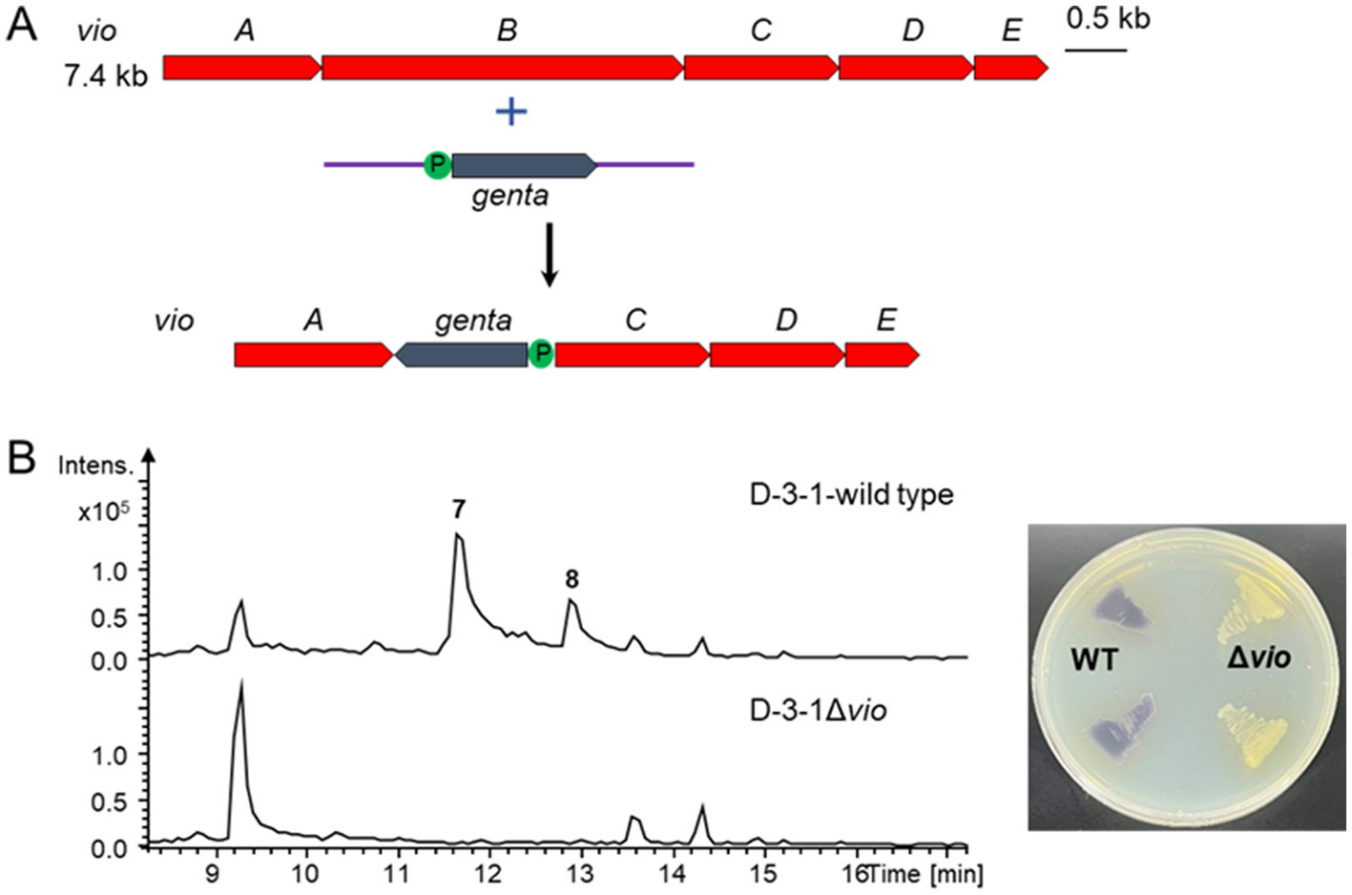
Knockout of violacein gene cluster in *Pseudoduganella* sp. D-3-1 based on recombination system Redγ-BAS and analysis of metabolic profiling. **(A)** Diagram of knockout of violacein gene cluster in *Pseudoduganella* sp. D-3-1. **(B)** HR-ESI-MS analysis of metabolic profile of wild type and Δ*vio* mutant at *m*/*z* 344 and *m*/*z* 328, and colony morphology between wild type and Δ*vio* mutant.

To investigate the metabolite production associated with the *pdb* BGC, we first established an efficient genome editing system in strain D-3-1. Three recombination systems—Redγ*βα*, Redγ-Redαβ7029, and Redγ-BAS—were evaluated for their ability to mediate homologous recombination. Each recombinase pair was individually introduced into strain D-3-1 via electroporation. A 3,032 bp fragment (1,908,943–1,911,975) within the *vio* gene cluster was targeted and replaced with a gentamicin resistance gene flanked by approximately 1 kb homology arms (Figure 4A). Successful recombinants were confirmed by colony PCR. Among the tested systems, only Redγ-BAS enabled genome modification, while Redγ*βα* and Redγ-Redαβ7029 were ineffective. Based on these results, Redγ-BAS was selected as the optimal recombination system for subsequent genome engineering in strain D-3-1.

**Figure 5:**
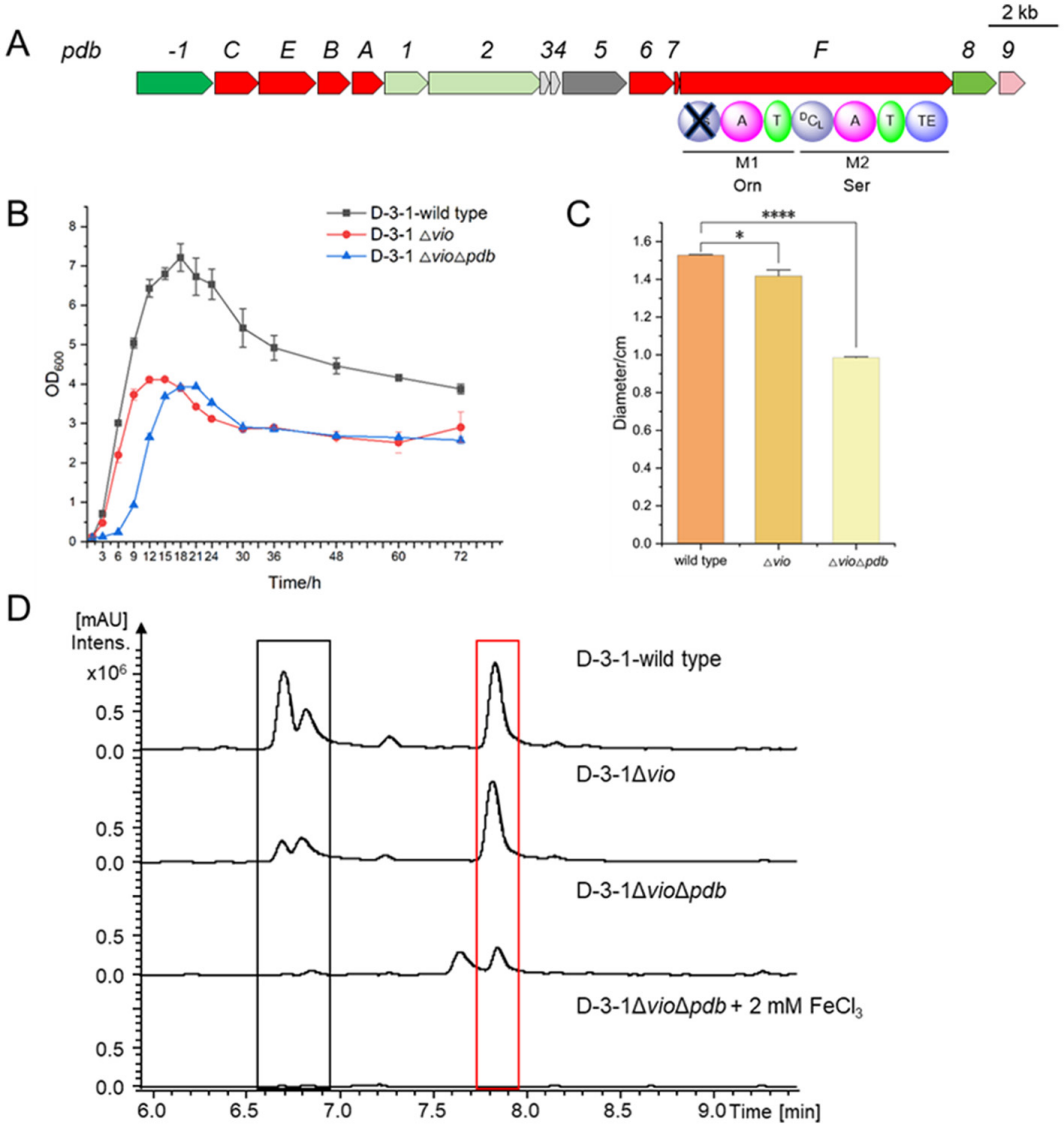
Knockout of pseudodubactin gene cluster in *Pseudoduganella* sp. D-3-1Δ*vio* and analysis of metabolic profiling and growth. **(A)** Diagram of knockout of *pdb* gene cluster in *Pseudoduganella* sp. D-3-1/pBBR1-Rha-Redγ-BAS-Km. **(B)** Growth curve of D-3-1 wild type, D-3-1Δ*vio* mutant, and D-3-1Δ*vio*Δ*pdb* mutant in 0238 medium condition. **(C)** Siderophore activity of crude extracts of D-3-1 wild type, D-3-1Δ*vio* mutant, and D-3-1Δ*vio*Δ*pdb* mutant. Data are presented as mean values ± SD, n = 3 biologically independent samples. All P values were determined by two-tailed unpaired t test. *p < 0.05, ****p < 0.0001. **(D)** HR-ESI-MS analysis of metabolic profile of D-3-1 wild type, D-3-1Δ*vio* mutant and D-3-1Δ*vio*Δ*pdb* mutant.

**Figure 6:**
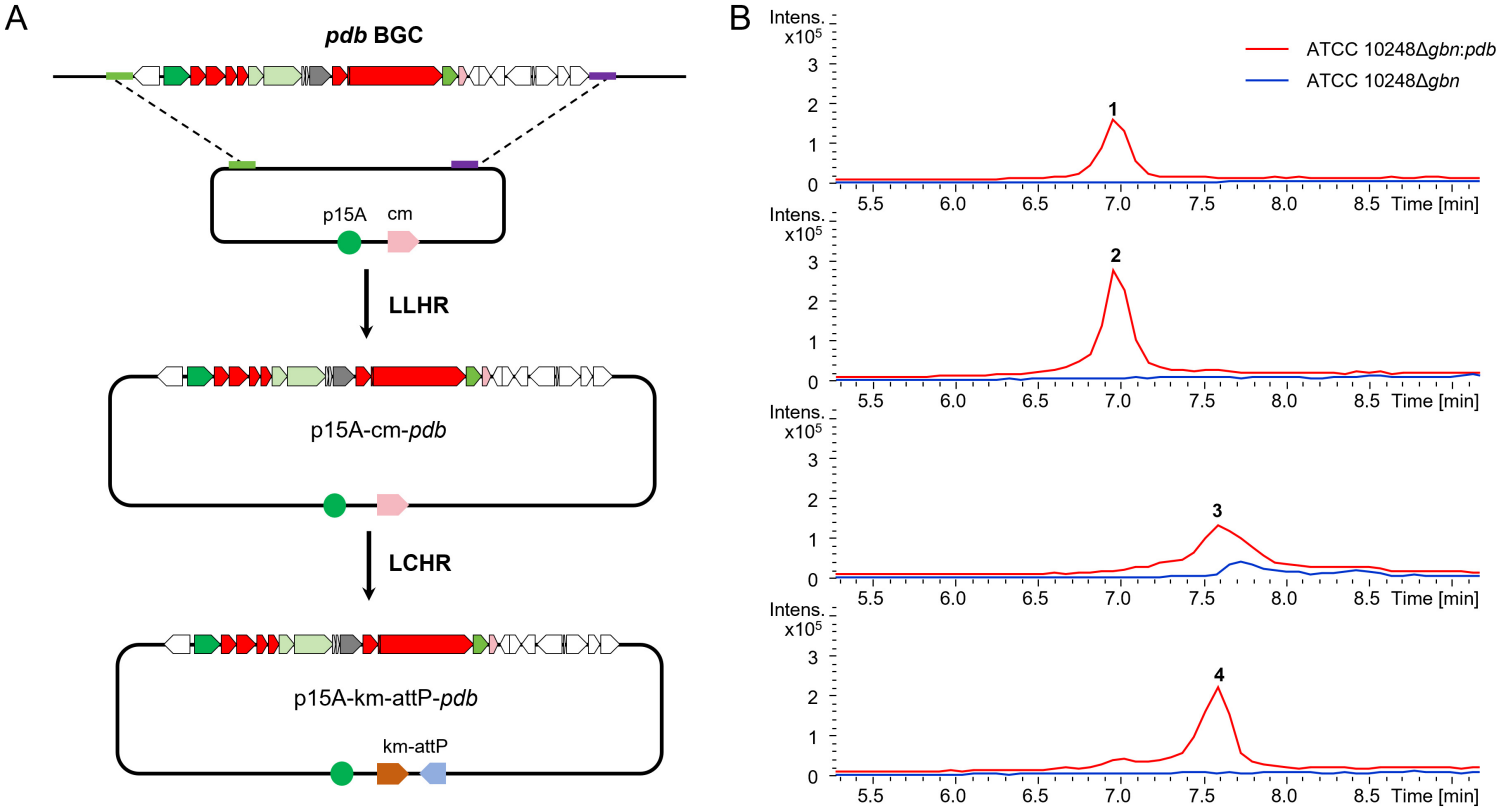
Heterologous expression of *pdb* BGC. **(A)** A diagram of direct cloning and modification of *pdb* BGC. LLHR: linear and linear homologous recombination; LCHR: linear and circle homologous recombination. The white color indicates other genes. The purple and green color line indicates homologous arms. **(B)** HR-ESI-MS analysis of crude extracts of wild-type strain (blue color) and recombinant carrying *pdb* BGC (red color).

By comparing colony morphology and metabolic profiles between the mutants and wild type strain, we confirmed the complete knockout of the *vio* BGC (Figure 4B). Subsequently, to investigate the role of the *pdb* cluster in siderophore biosynthesis, the starter condensation (Cs) domain in core gene *pdbF* was replaced with an apramycin resistance gene (Figure 5A), and recombinants were again verified by colony PCR. HR-ESI-MS analysis of the double mutant strain D-3-1Δ*vio*Δ*pdb* mutant revealed a complete loss of turnerbactin analogs (**1-4**), while compounds **5-6** remained detectable, albeit at reduced levels compared to the wild type and D-3-1Δ*vio* strains (Figure 5D and Supplementary Figure 4). In addition, we cloned the *pdb* cluster and transferred it into heterologous host *B. gladioli* ATCC 10248Δ*gbn*-attB to confirmed if the *pdb* cluster biosynthesizing **1-4**. HR-ESI-MS/MS analysis result revealed the target compounds **1-4** were detected in recombinant *B. gladioli* ATCC 10248Δ*gbn*:*pdb* (Figure 6). These results suggest that the *pdb* gene cluster is responsible for the biosynthesis of compounds **1–4** but not required for the production of compounds **5** and **6**. However, the absence of compounds **1–4** appears to influence the production levels of compounds **5** and **6**.

Growth assays showed that both D-3-1Δ*vio* and D-3-1Δ*vio*Δ*pdb* mutants exhibited slower growth compared to the wild type strain. Moreover, siderophore activity was markedly reduced in the crude extract of the double mutant (Figures 5B, 5C). These findings confirm that violacein contributes to the growth of *Pseudoduganella* sp. D-3-1, and that the *pdb* gene cluster is responsible for the biosynthesis of siderophore compounds **1–4**.

### 3.5 Pseudodubactin biosynthetic gene cluster in *Pseudoduganella* sp. D-3-1

**Figure 7:**
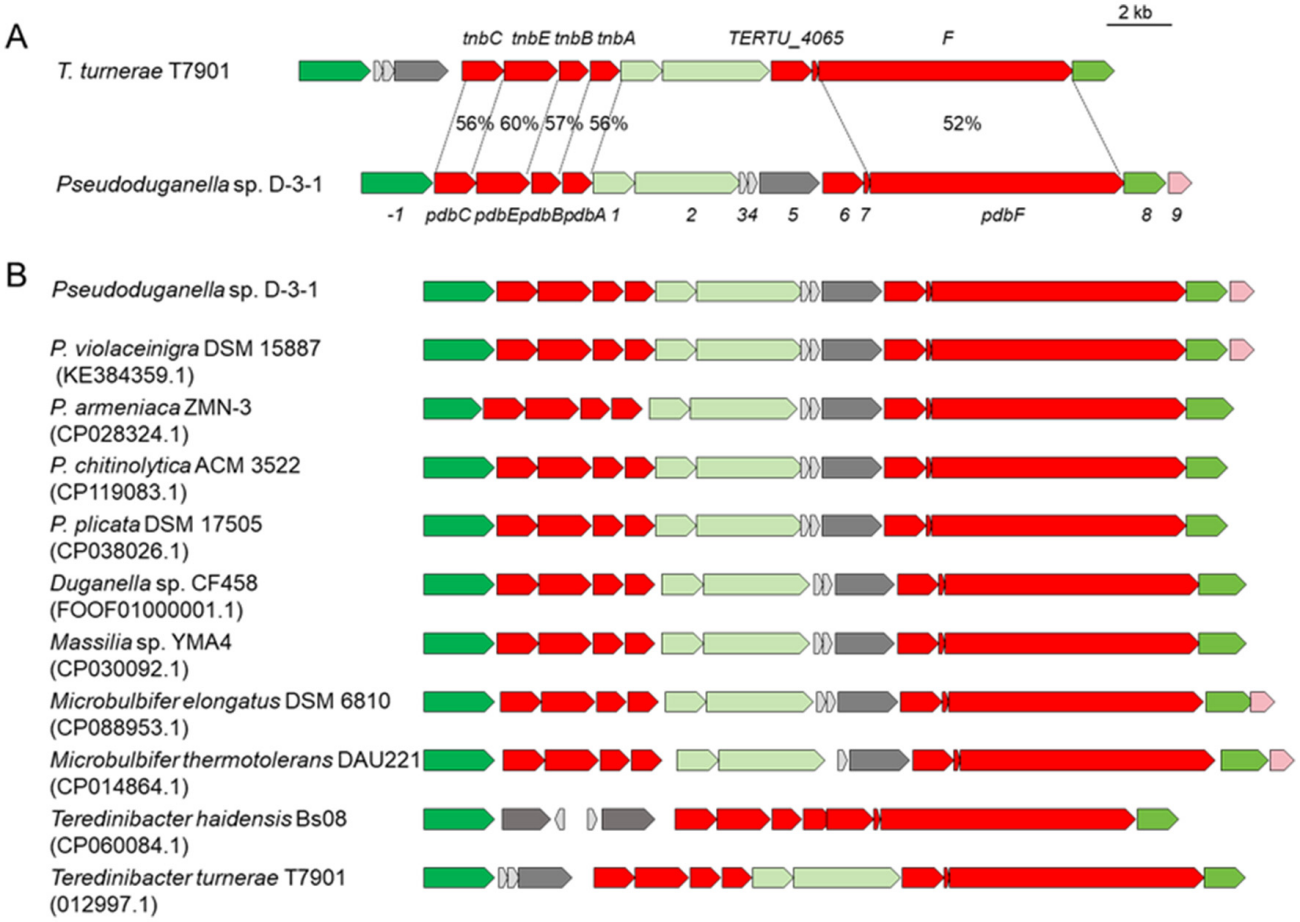
Analysis of *pdb* BGC. **(A)** Comparison between predicted *pdb* BGC from *Pseudoduganella* sp. D-3-1 and the similar *tnb* BGC from *T. turnerae* T7901. *-1*: TonB-dependent siderophore receptor; *pdbC*: Isochorismate synthase; *pdbE*: 2,3-dihydroxybenzoate-AMP ligase; *pdbB*: Isochorismatase; *pdbA*: 2,3-dihydroxybenzoate-2,3-dehydrogenase; *pdbF*: NRPS; 1: RND family efflux transporter; 2: AcrB/AcrD/AcrF family protein; 3 and 4: hypothetical protein; 5: PepSY-associated TM helix domain-containing protein; 6: enterochelin esterase; 7: MbtH-like protein; 8: major facilitator transporter; 9:4′-phosphopantetheinyl transferase. **(B)** Comparative analysis with pseudodubactin biosynthetic gene clusters of different strains.

Genome annotation of *Pseudoduganella* sp. D-3-1 revealed a NRPS gene cluster, designated as *pdb* gene cluster, exhibiting moderate similarity to the known *tnb* BGC, responsible for turnerbactin production. Comparative analysis showed that the core protein PdbCEBA and PdbF are homologous to TnbCEBA and TnbF, respectively, and the other accessory proteins also displayed high homolog (Figure 7A and Supplementary Table 3).

**Figure 8:**
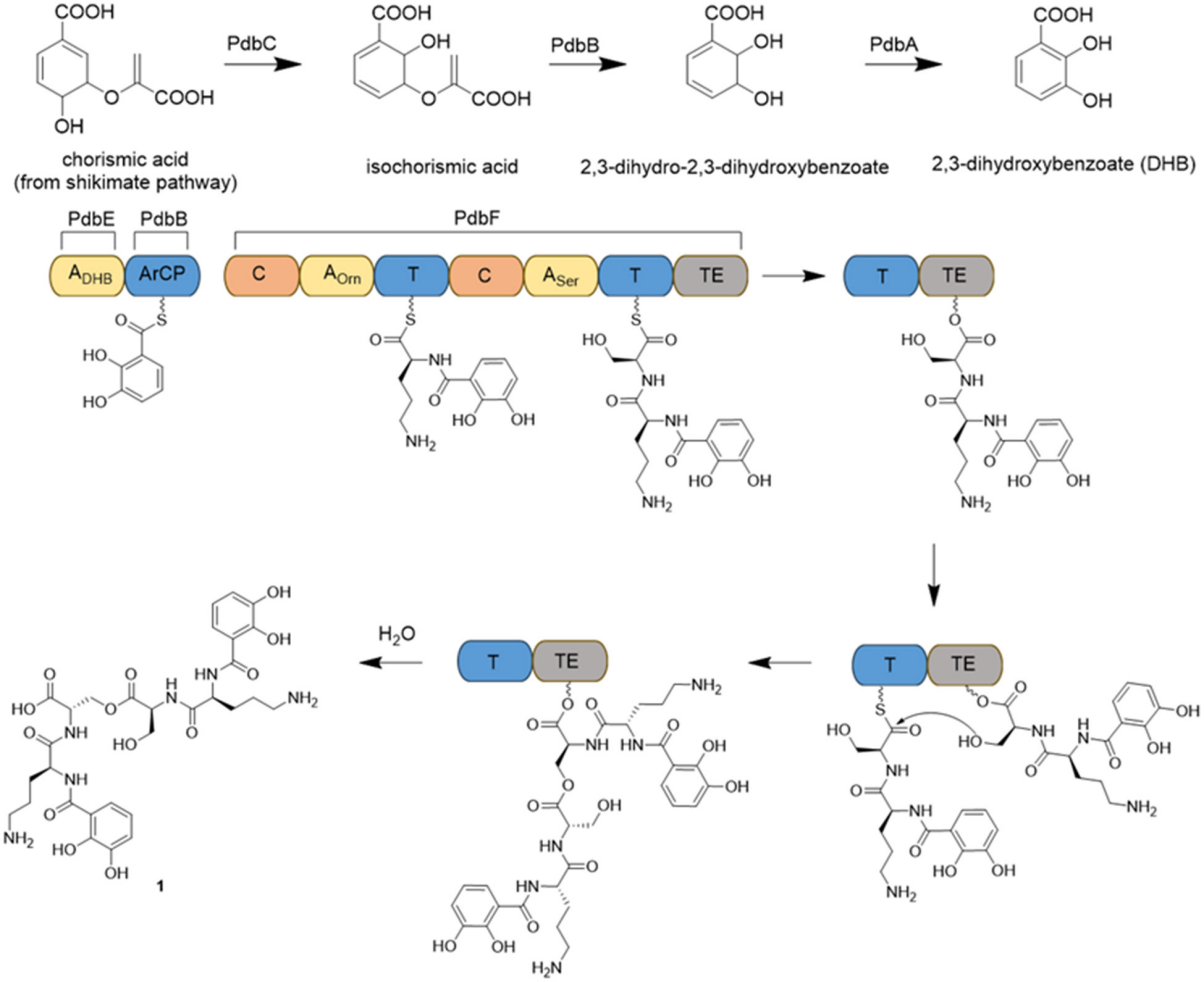
Proposed pathways for 2,3-Dihydroxybenzoic Acid (DHB) biosynthesis and pseudodubactin biosynthesis. C: Condensation domain A, Adenylation domain; T, Thiolation domain; TE: Thioesterase domain.

Based on established biosynthetic pathways for turnerbactin and enterobactin (Gehring et al., 1997; Dahm et al., 1998; Han et al., 2013; Amiri et al., 2025), we propose that *pdbCEBA* are responsible for the biosynthesis and activation of 2,3-dihydroxybenzoate (DHB) via the shikimate pathway (Figure 8). Specifically, isochorismate synthase PdbC catalyzes the isomerization of chorismate to isochorismate. This intermediate is then hydrolyzed by PdbB to form 2,3-dihydro-DHB, which is subsequently oxidized to DHB by PdbA. The enzyme PdbE then activates DHB and transfers it to PdbB for incorporation into the growing peptide. The NRPS enzyme PdbF comprises a two-module assembly line with the domains Cs–A_1_-T–^D^C_L_-A_2_-T–TE. The adenylation domains A_1_ and A_2_ are specific for ornithine (Orn) and serine (Ser), respectively, via bioinformatic predictions. The Cs domain facilitates the coupling of DHB to Orn, initiating siderophore assembly. Thus, the Orn residue of turnerbactin analogs is D configure. Additionally, gene 7 encodes an MbtH-like protein (Miller et al., 2016), known to enhance the activity of adenylation domain, and gene 9 encodes a 4′-phosphopantetheinyl transferase involved in the activation of thiolation (T) domains (Mofid et al., 2004). Notably, this phosphopantetheinyl transferase is absent in the *tnb* gene cluster, suggesting a divergence in the biosynthetic assembly line between the *pdb* and *tnb* systems.

The *pdb* BGC is reported here for the first time in a bacterial strain of the order *Burkholderiales*. Comparative genomic analysis revealed that the *pdb* cluster is conserved across multiple *Pseudoduganella* species, including *P. armeniaca, P. plicata, P. chitinolytica*, and *P. violaceinigra*. It was also detected in other strains, such as *Dugnella, Massilia, Microbulbifer*, and *Teredinibacter* (Figure 7B). Moreover, a similar *tnb*-like gene cluster has been identified in *Pseudomonas psychrotolerans* PRS08-11306, where it has been implicated in nitrogen fixation and plant association (Liu et al., 2017), as well as *Pseudomonas* sp. 102515 (Fidan and Zhan, 2019) and *P. oryzihabitans* RIT370 (Gan et al., 2015). These findings suggest that the conserved *pdb* BGC may play important ecological roles in microbial interactions or plant-microbe symbiosis, which warrants further investigation.

## 4 Discussion

Strain D-3-1 is preliminarily classified as *Pseudoduganella* sp. based on combined 16S rRNA gene and whole-genome sequence analyses in this work. *Pseudoduganella* sp. D-3-1 is a bacterium with antibacterial and siderophore activities, belonging to the order *Burkholderiales*. The genus *Pseudoduganella* is rarely reported, with only 21 taxa documented (https://lpsn.dsmz.de/genus/Pseudoduganella), and relevant studies are currently limited. *Burkholderia* and *Paraburkholderia* are two representative genera of *Burkholderiales* widely used as biocontrol agents, particularly owing to their production of diverse antifungal metabolites and siderophores (Mannaa et al., 2018; Elshafie and Camele, 2021). The *Pseudoduganella* strain identified in this study represents a novel candidate for biocontrol applications. Genome-based biosynthetic gene cluster prediction revealed that this strain harbors multiple gene clusters responsible for unknown secondary metabolites and siderophores. Therefore, the development of *Pseudoduganella* as a biocontrol bacterium extends the agricultural applications of other genera within the order *Burkholderiales*.

*Pseudoduganella* sp. D-3-1 can synthesize at least two types of siderophores, comprising a catecholate-type turnerbactin analog and uncharacterized siderophores, along with the bioactive metabolite violacein. As an L-serine-derived catecholate siderophore, turnerbactin was first characterized in *Teredinibacter turnerae* and naturally occurs as linear monomers, dimers and trimers (Han et al., 2013). In this study, fermentation extracts of strain D-3-1 contained dimeric, dehydrodimeric turnerbactin analogs, together with DHB-Orn-Ser-Ser and dehydro-DHB-Orn-Ser-Ser, while linear trimer turnerbactin was absent. Heterologous expression revealed that the *pdb* gene cluster exclusively biosynthesized linear dimers in *B. gladioli* ATCC 10248Δ*gbn*, and the absence of turnerbactin in the native strain was confirmed to be independent of purification artifacts. A similar polymeric pattern is observed in vanchrobactin from *Vibrio campbellii* DS40M4, which produces monomeric, dimeric and trimeric siderophore congeners (Sandy et al., 2010). Combined with the high sequence homology between *pdb* and *tnb*, it is proposed that linear dimers represent the genuine natural products of the *pdb* BGC.

The dehydrated serine residue within dehydrated (DHB-Orn-Ser) is commonly found in numerous microbial secondary metabolites, including thiamyxin, AM-toxin, microcystin and albopeptide (Wang et al., 2024). *In vitro* assays have validated that C_modAA_ domains are capable of catalyzing serine dehydration. For instance, the C5 domain from the nocardicin biosynthetic cluster mediates the conversion of serine to dehydroalanine (Dha), which contributes to β-lactam skeleton formation (Wheadon and Townsend, 2021). Likewise, during albopeptide biosynthesis, the AlbB-C2 and AlbB-C3 domains catalyze the dehydration of serine and threonine to generate Dha and *E*-Dhb, respectively (Wang et al., 2021). In microcystin, the formation of N-methyl-dehydrated serine is tentatively attributed to the unvalidated dehydrogenase McyJ (Tillett et al., 2000). Nevertheless, genomic analysis revealed that PdbF lacks a functional C domain, and no McyJ homolog was identified in strain D-3-1 to drive serine dehydration. Accordingly, the enzymatic mechanism responsible for Dha formation in dehydrated (DHB-Orn-Ser) remains ambiguous and requires further exploration.

The new linear analogs **2** and **4** are postulated to be hydrolytic derivatives of compounds **1** and **3**, respectively. At present, their chemical structures remain only preliminarily assigned based on MS/MS fragmentation pattern matching with known turnerbactin analogs, owing to the lack of definitive NMR spectroscopic evidence. Notably, these two analogs exhibit low biosynthetic yields and highly similar polarity to their parental compounds **1** and **3**. Their high structural similarity and strong polarity hinder chromatographic separation and high-purity purification, limiting their full structural characterization in this study. Future efforts will optimize chromatographic protocols to purify sufficient quantities of analogs **2** and **4** for comprehensive NMR-based structural elucidation. Complete structural determination of these novel analogs will lay a foundation for deciphering the biosynthetic pathway of *pdb*-encoded turnerbactin derivatives. Two additional novel siderophores (compounds **5** and **6**) differ structurally from turnerbactin analogs. MS/MS fragmentation analysis demonstrated that the fragmentation patterns of compounds **5** and **6** do not match the typical fragment signatures of NRPS-derived metabolites. Accordingly, bioinformatic analysis failed to identify the gene cluster responsible for their biosynthesis. Further genome mining uncovered six TonB-dependent siderophore receptors, which are universally involved in the uptake and transmembrane transport of siderophores (Noinaj et al., 2010). Future studies focus on the definitive structural resolution of compounds **5** and **6** to characterize their unique functional moieties. Combined with enzyme mining strategies and in-frame knockout of TonB-dependent receptor genes, these efforts will facilitate the identification of the novel biosynthetic gene cluster governing the production of the two uncharacterized siderophores.

The *pdb* BGC is widely distributed across β-proteobacteria (*Pseudoduganella, Massilia, Duganella*) and γ-proteobacteria (*Microbulbifer, Teredinibacter, Pseudomonas*). Functionally characterized *tnb*-like clusters in *Pseudomonas* strains are involved in nitrogen fixation and plant association, implying conserved ecological functions of homologous *pdb* BGC. Furthermore, *pdb* knockout remarkably reduced the production of the uncharacterized siderophore. Collectively, the conserved *pdb* BGC serves as a vital regulator in siderophore biosynthesis and may modulate microbial interactions and plant-microbe symbiosis. Further biochemical and phenotypic experiments are necessary to elucidate its biosynthetic mechanism, specific metabolites, and ecological significance.

In this study, we successfully characterized a novel strain, *Pseudoduganella* sp. D-3-1, which exhibits strong antifungal activity and siderophore production. A functional genetic recombination system was established to enable the targeted knockout of the *vio* and *pdb* BGCs. Four turnerbactin analogs were identified as metabolites synthesized by the *pdb* BGC, marking the first report of such compounds in the genus *Pseudoduganella*. Integrative analyses combining genome mining, MS-based metabolite profiling, gene knockout, and heterologous expression experiments revealed that *Pseudoduganella* sp. D-3-1 harbors a diverse repertoire of secondary metabolites, with siderophores serving as the dominant bioactive components. Collectively, this work provides the first comprehensive insight into the biosynthetic potential of a *Pseudoduganella* strain and highlights its promise as a novel biocontrol agent, laying the groundwork for future applications in sustainable agriculture and biotechnology.

## Data Availability

The genome sequence of strain D-3-1 reported here was deposited in GenBank at NCBI under the BioProject accession number PRJNA1330495 and Biosample accession number SAMN51523887.
