# Supplementary material for: Complete genome sequence and mass spectrometry assist mining siderophore turnerbactin analogs in a non-model Pseudoduganella strain with biocontrol potential
Source: Front Microbiol. 2026 Jul 23;17:1888443. doi: 10.3389/fmicb.2026.1888443 (PMC13442633; doi:10.3389/fmicb.2026.1888443)
Supplement: Supplementary file 1 [file Supplementary_file_1.docx]

Supplementary Material

# 1 Contents

Table S1. Strains, mutants and plasmids used in this study

Table S2. Primers used in this study

Table S3. Protein identity between *pdb* and *tnb* biosynthetic gene clusters

Figure S1. Genome annotation based on public databases

Figure S2. HR-ESI-MS analysis of the metabolic profile of *Pseudoduganella* sp. D-3-1 in iron-limited conditions at EIC 693, 390, 169.

Figure S3. HR-ESI-MS analysis of violacein and deoxyviolacein produced by *Pseudoduganella* sp. D-3-1 in iron-limited and iron-replete conditions and their structures.

Figure S4. HR-ESI-MS analysis of metabolic profile of wild type and mutants at *m/z* 169 and *m/z* 390.

# 2 Supplementary Tables

## 2.1 Supplementary Table S1. Strains, mutants and plasmids used in this study

| Strains/plasmids | Description | Source |
| --- | --- | --- |
| *Pseudoduganella* sp. D-3-1 | Wild type | This study |
| D-3-1Δ*vio* | Knockout of *vio* gene cluster, a 3,032 bp fragment (1,908,943–1,911,975) was replaced by *genta* | This study |
| D-3-1Δ*vio*Δ*pdbF* | Knockout of *vio* and *pdb* gene clusters, a 3,032 bp fragment (1,908,943–1,911,975) was replaced by *genta* and a Cs domain in *pdbF* was replaced by *apra* | This study |
| pBBR1-Rha-Redγβα-Km | BR322 replicon, km^R^, containing Redγβα system | Zheng et al., 2020, Chen et al., 2021 |
| pBBR1-Rha-Redγ-BAS-Km | BR322 replicon, km^R^, containing Redγ-BAS system | Zheng et al., 2020, Chen et al., 2021 |
| pBBR1-Rha-Redγ-Redαβ7029-Km | BR322 replicon, km^R^, containing Redγ-Redαβ7029 system | Zheng et al., 2020, Chen et al., 2021 |

## 2.2 Supplementary Table S2. Primers used in this study

| **Primers** | **Primer sequences（5’-3’）** |
| --- | --- |
| 27F | AGAGTTTGATCCTGGCTCAG |
| 1492R | GGTTACCTTGTTACGACTT |
| Km-check-S | GAGAGGCTATTCGGCTATGA |
| Km-check-A | CAAGCTCTTCAGCAATATCAC |
| apra-primer-S | ACGCTCAGTGGAACGAGGTT |
| apra-primer-A | AATCTGTACCTCCTTAAGTCAG |
| PseBGC14KO-HA11-S | AGAGAAGGAATGGGTGTGCG |
| PseBGC14KO-HA11-A | AACCTCGTTCCACTGAGCGTGCAGGTAGGGGACGCTATTT |
| PseBGC14KO-HA22-S | CTGACTTAAGGAGGTACAGATTCGAAGGGGTTGATGGCGA |
| PseBGC14KO-HA22-A | GCTTGAGACCAATGCCCAG |
| 14KO-150HA-primer-S | GCCAGCGCCAGCGCCTGCA |
| 14KO-150HA-primer-A | TCACCGTGCTGCGCAACGACG |
| vioKO-HA1-S | CGAGCAGCAGGAATTGAAGG |
| vioKO-HA1-A | CATTTAAGTTGCAGGTCGTTTCGCCGTCATCGCCTTCTTCATCG |
| Genta-primer-S | GCGAAACGACCTGCAACTTAAATG |
| Genta-primer-A | TACGCGCCCGGGGAGCCC |
| vioKO-HA2-S | CCTTGGGCTCCCCGGGCGCGTAGGGGAACGATGTCTAGAGAGG |
| vioKO-HA2-A | CGCCATATTCATGCCCTGTC |
| Apra-check-S | CTTCTCCTCGGCGTGGGACA |
| Apra-check-A | TGTCCCACGCCGAGGAGAAG |
| PseBGC14-clone-S1 | GCCCAGCCAGCGTCAACCTGACCTCGCCAAGCAAATTGGATCTTAAGGATCTCCAGGCA |
| PseBGC14-clone-A1 | GGCGGCTGGAAAGGCGACTAAGGAGCGCTGCCAGGGGAAGCCTTCTTATTCGGCCTTG |
| PseBGC14-clone-S2 | GTGCAACCCTTCGATATAGTGAGCTGTGTAAGGCCCAGCCAGCGTCAACCT |
| PseBGC14-clone-A2 | GCGCCAGCGTGGACCTGTTCCGGGCGCTGGGCGGCGGCTGGAAAGGCGACTA |
| short-attP-S | CTGGATCTATCAACAGGA |
| short-attP-A | GATTAGAAAAACAACTTAAATG |
| tnb-check-S1 | TCATAGCAGCCGATCTCCAG |
| tnb-check-A1 | GCTTGAGACCAATGCCCAG |
| tnb-check-S2 | CACATATCGTTCAGCAGGGC |
| tnb-check-A2 | AAGTGGAAAACCTGCTGCTG |

## 2.3 Supplementary Table S3. Protein identity between *pdb* and *tnb* biosynthetic gene clusters

| **Gene** | **Product** | **Identity (*T. turnerae* T7901)** |
| --- | --- | --- |
| *-1* | TonB-dependent receptor domain-containing protein | 50.96% (317/622) |
| *pdbC* | isochorismate synthase | 56.08% (203/362) |
| *pdbE* | 2,3-dihydroxybenzoate-AMP ligase | 59.77% (318/532) |
| *pdbB* | isochorismatase | 57.19% (167/292) |
| *pdbA* | 2,3-dihydroxybenzoate-2,3-dehydrogenase | 55.69% (142/255) |
| *1* | Multidrug efflux pump subunit AcrA | 41.19% (159/386) |
| *2* | Multidrug efflux pump subunit AcrB | 60.29% (618/1025) |
| *3* | hypothetical protein | 24.69% (20/81) |
| *4* | hypothetical protein | 57.89% (55/95) |
| *5* | PepSY-associated TM helix domain-containing protein | 52.74% (289/548) |
| *6* | Enterochelin esterase | 39.06% (166/425) |
| *7* | MbtH-like protein | 51.67% (31/60) |
| *pdbF* | NRPS | 52.41% (1263/2410) |
| *8* | Enterobactin exporter | 61.76% (252/408) |
| *9* | 4'-phosphopantetheinyl transferase | - |

## 2.4 Supplementary Table S4 Phylogenetic relationships of strain D-3-1 with its closest related species

|  | Strain D-3-1 | | |
| --- | --- | --- | --- |
|  | 16S rRNA identities | ANI (%) | dDDH (%) |
| *P. violaceinigra* DSM 15887^T^ | 98.9% | 88.2% | 36.6% |
| *P. danionis* DSM 103461^T^ | 98% | 75.% | 21.3% |
| *P.* *umbonate* CECT 7753^T^ | 98% | 74.4% | 22.3% |
| *P. lutea* DSM 17473^T^ | 98% | 76% | 22.2% |

Note: DSM 15887^T^ (=YIM 31327^T^) (Kämpfer et al, 2012), DSM 103461^T^ (=E3/2^T^) (Kämpfer et al, 2016), DSM 26121^T^ (=LP01^T^) (Lu et al, 2022) and DSM17473^T^ (=101^T^) (Lu et al, 2022)

# 3 Supplementary Figures

## 3.1 Supplementary Figure S1. Genome annotation based on public databases. (A) Nr homologous species distribution. (B) The eggNOG function classification of consensus sequence. (C) Circos plot exhibiting the genomic features of *Pseudoduganella* sp. D-3-1.

## 3.2 Supplementary Figure S2. HR-ESI-MS analysis of the metabolic profile of *Pseudoduganella* sp. D-3-1 in iron-limited conditions at EIC 693, 390, 169.

## 3.3 Supplementary Figure S3. HR-ESI-MS analysis of violacein and deoxyviolacein produced by *Pseudoduganella* sp. D-3-1 in iron-limited and iron-replete conditions and their structures.

## 3.4 Supplementary Figure S4. HR-ESI-MS analysis of metabolic profile of wild type and mutants at *m/z* 169 and *m/z* 390

**References**

Zheng, W. T., Wang, X., Zhou, H. B., Zhang, Y. M., Li, A. Y. and Bian, X. Y. (2020). Establishment of recombineering genome editing system in *Paraburkholderia megapolitana* empowers activation of silent biosynthetic gene clusters. *Microb. Biotechnol*. 13(2):397-405. doi:10.1111/1751-7915.13535

Chen, H. N., Sun, T., Bai, X. P., Yang, J., Yan, F., Yu, L., et al. (2021). Genomics-driven activation of silent biosynthetic gene clusters in *Burkholderia gladioli* by screening recombineering system. *Molecules*. 26(3): 700. doi: 10.3390/molecules26030700

Kampfer, P., Wellner, S., Lohse, K., Martin, K. and Lodders, N. (2012). *Duganella phyllosphaerae* sp. nov., isolated from the leaf surface of *Trifolium repens* and proposal to reclassify *Duganella violaceinigra* into a novel genus as *Pseudoduganella violceinigra* gen. nov., comb. nov. *Syst. Appl. Microbiol.* 35(1): 19-23. doi: 10.1016/j.syapm.2011.10.003

Kämpfer, P., Irgang, R., Busse, H. J., Poblete-Morales, M., Kleinhagauer, T., Glaeser S. P., et al. (2016) *Pseudoduganella danionis* sp. nov., isolated from zebrafish (Danio rerio). Int J Syst Evol Microbiol. 66(11):4671-4675. doi: 10.1099/ijsem.0.001408

Lu, H. B., Song, D., Deng, T. C., Mei, C. F., Xu, M. Y. (2022) *Duganella vulcania* sp. nov., *Rugamonas fusca* sp. nov., *Rugamonas brunnea* sp. nov. and *Rugamonas apoptosis* sp. nov., isolated from subtropical streams, and phylogenomic analyses of the genera *Janthinobacterium*, *Duganella, Rugamonas*, *Pseudoduganella* and *Massilia*. Int J Syst Evol Microbiol. 72(9). doi: 10.1099/ijsem.0.005407
